# Role of GABA_A_-Mediated Inhibition and Functional Assortment of Synapses onto Individual Layer 4 Neurons in Regulating Plasticity Expression in Visual Cortex

**DOI:** 10.1371/journal.pone.0147642

**Published:** 2016-02-03

**Authors:** Ignacio Saez, Michael J. Friedlander

**Affiliations:** Virginia Tech Carillion Research Institute, 2 Riverside Circle, Roanoke, Virginia 24016, United States of America; University of Michigan, UNITED STATES

## Abstract

Layer 4 (L4) of primary visual cortex (V1) is the main recipient of thalamocortical fibers from the dorsal lateral geniculate nucleus (LGN_d_). Thus, it is considered the main entry point of visual information into the neocortex and the first anatomical opportunity for intracortical visual processing before information leaves L4 and reaches supra- and infragranular cortical layers. The strength of monosynaptic connections from individual L4 excitatory cells onto adjacent L4 cells (unitary connections) is highly malleable, demonstrating that the initial stage of intracortical synaptic transmission of thalamocortical information can be altered by previous activity. However, the inhibitory network within L4 of V1 may act as an internal gate for induction of excitatory synaptic plasticity, thus providing either high fidelity throughput to supragranular layers or transmittal of a modified signal subject to recent activity-dependent plasticity. To evaluate this possibility, we compared the induction of synaptic plasticity using classical extracellular stimulation protocols that recruit a combination of excitatory and inhibitory synapses with stimulation of a single excitatory neuron onto a L4 cell. In order to induce plasticity, we paired pre- and postsynaptic activity (with the onset of postsynaptic spiking leading the presynaptic activation by 10ms) using extracellular stimulation (ECS) in acute slices of primary visual cortex and comparing the outcomes with our previously published results in which an identical protocol was used to induce synaptic plasticity between individual pre- and postsynaptic L4 excitatory neurons. Our results indicate that pairing of ECS with spiking in a L4 neuron fails to induce plasticity in L4-L4 connections if synaptic inhibition is intact. However, application of a similar pairing protocol under GABA_A_Rs inhibition by bath application of 2μM bicuculline does induce robust synaptic plasticity, long term potentiation (LTP) or long term depression (LTD), similar to our results with pairing of pre- and postsynaptic activation between individual excitatory L4 neurons in which inhibitory connections are not activated. These results are consistent with the well-established observation that inhibition limits the capacity for induction of plasticity at excitatory synapses and that pre- and postsynaptic activation at a fixed time interval can result in a variable range of plasticity outcomes. However, in the current study by virtue of having two sets of experimental data, we have provided a new insight into these processes. By randomly mixing the assorting of individual L4 neurons according to the frequency distribution of the experimentally determined plasticity outcome distribution based on the calculated convergence of multiple individual L4 neurons onto a single postsynaptic L4 neuron, we were able to compare then actual ECS plasticity outcomes to those predicted by randomly mixing individual pairs of neurons. Interestingly, the observed plasticity profiles with ECS cannot account for the random assortment of plasticity behaviors of synaptic connections between individual cell pairs. These results suggest that connections impinging onto a single postsynaptic cell may be grouped according to plasticity states.

## Introduction

Primary visual cortex (V1) receives visual information from the retina via incoming thalamocortical axons, which target primarily neurons within L4 [1,2]. Axonal projections arising from L4 excitatory cells target mostly adjacent L4 cells as well as excitatory pyramidal neurons located in L2/3, in the first stage of cortical processing of visual information [3]. Synaptic plasticity in sensory cortices contributes to a variety of important functions including sensory map reorganization and refinement during normal development [4–6], functional reorganization in response to imbalanced early sensory experience or injury [7–9] and perceptual learning [10,11]. Both sets of projections arising from L4 excitatory neurons (intralaminar L4-L4 and ascending interlaminar L4-L2/3 projections) have been demonstrated to be important loci of synaptic plasticity [12,13], and may contribute to these processes. L4-L4 intralaminar connections account for the majority of excitatory synapses within L4 [14], so it is of particular interest to understand the characteristics of plasticity induction and expression in these synapses. Furthermore, the activation of the inhibitory network within L4 is a critical gate for plasticity induction in supragranular layers [15], but it is unknown whether it also limits intralaminar plasticity within L4.

Using extracellular stimulation techniques, we have previously shown that bouts of paired pre- and postsynaptic activity are effective at inducing plasticity in connections from L4 onto L2/3 pyramidal cells [16], and that the sign of this plasticity outcome is variable-in some cases, pairing results in long-term potentiation (LTP), in some cases in long-term depression (LTD) and in some cases in no change (NC) of synaptic strength. The same is true for unitary L4-L4 connections [17], which raises the question whether overall plasticity occurs when multiple afferents onto L4 cells, which might undergo plasticity of opposite signs, are simultaneously paired. To answer this question we used extracellular stimulation (ECS, as opposed to single cell stimulation, SCS) of multiple L4 afferents onto single postsynaptic cells while recording the evoked responses in the individual postsynaptic neuron before and after pairing of pre- and postsynaptic activity. Consistent with the hypothesis that an inhibitory gate controls synaptic plasticity in L4 [12,18], if inhibition is intact, little or no plasticity was elicited in response to pairing. However, if the GABA_A_R antagonist bicuculline was present in the bath (2μM), individual cells underwent significant LTP, LTD or NC in response to ECS pairing. These results suggest that inhibitory circuitry limits the expression of synaptic plasticity within L4 and may be a mechanism whereby intralaminar L4-L4 connections remain stable.

## Methods

### Slice preparation

All experiments were performed according to guidelines and approved by the Institutional Animal Care and Use Committees (IACUC) of the Virginia Tech Carilion Research Institute. Tri-color guinea pigs of ages p6-14 were deeply anesthetized with a mixture of 0.85mg/kg ketamine and 0.15mg/kg xylazine and decapitated. The brain was rapidly removed and cooled for at least 90 seconds in artificial cerebrospinal fluid (aCSF) containing (in mM) 124 NaCl, 2 KCl, 2 MgSO_4_, 2 CaCl_2_, 1.25 KH_2_PO_4_, 26 NaHCO_3_, and 11 dextrose, and saturated with 95% O_2_/5% CO_2_ to a final pH of 7.4. Coronal slices of the visual cortex were cut at 300 um with a Vibratome 1000 Plus (Technical Products International). Slices were incubated at 33–35°C for 45–60 min in a holding chamber in a heated water bath (Fisher Scientific) and then transferred to a room temperature bath until being transferred to a submerged recording chamber (Warner Instruments) and perfused continuously at 2–3 ml/min with oxygenated aCSF at 32–34°C. Neurons were visualized with a Zeiss upright microscope (Axioskope FS1; Zeiss) equipped with an Achroplan 40x 0.8 numerical aperture water immersion lens set up for Differential Interference Contrast (DIC) microscopy.

Glass micropipettes [Corning 7056 glass (1.5 OD, 1.12 ID); A-M Systems, Carlsborg, WA] were pulled on a vertical puller (PP-830; Narishige) to an open tip resistance of 2.5–4.0 MΩ and filled with a pipette solution containing (in mM) 115 K-gluconate, 20 KCl, 10 HEPES, 4 NaCl, 4 Mg-ATP, 0.3 Na-GTP, and 4 Phosphocreatine-Na, with the pH adjusted to 7.4 by KOH. Osmolarity was adjusted to 280–290 mOsm with mannitol.

### Electrophysiology

All recordings were made with a MultiClamp 700B amplifier (Molecular Devices), and signals were digitized at 20 kHz with a Digidata digitizer 1440A and recorded using Clampex 9 or 10 software (Molecular Devices). Recordings were filtered on-line at 4 kHz with a four-pole Bessel low-pass filter. Layer 4 was identified under light and DIC microscopy in base to its differential opacity to transmitted light and the smaller size of L4 somata compared to L5 cells. Cells with membrane potentials more positive than –60 mV and recordings with high access resistance (>40 MΩ or >20% the value of the input resistance for that cell) were discarded. Once the whole-cell recording was established, the intrinsic firing properties of the patched cell were tested by injecting a 100 ms depolarizing current pulse; neurons that did not exhibit regular spiking typical of excitatory cells were considered as putative inhibitory neurons and discarded. For extracellular stimulation experiments, a concentric or bipolar electrode was placed in L4 laterally to the patched cell (typically 100–150μm). Cells that showed regular spiking with spike frequency adaptation were held in current-clamp (I-clamp) or voltage clamp (V-clamp). Subsequently, the extracellular stimulation intensity was adjusted (typically 20–30μA) until a response of about 3-5mV (under I-clamp; n = 14) or 350-400pA (under V-clamp; n = 14) was obtained. The synaptic response at this level of stimulation was then evoked at 0.2Hz for at least 10 minutes during which a stable baseline response was established as evaluated by linear fitting the time plot of the response; if a significant tendency (p<0.05) was observed, the cell was discarded. In a subset of experiments, slices were pre-incubated for >1h in aCSF containing 2μM bicuculline (Tocris Bioscience) to block GABA_A_-mediated inhibitory synaptic transmission. The bicuculline containing aCSF was also used to perfuse the slice during the recording (<2h recording time per slice). These experiments were performed in V-clamp (n = 27). A total of 32 recordings were discarded due to quality control (input resistance [R_i_] changes, unstable baseline, excessive access resistance; 9 in I-Clamp and 11 in V-Clamp without bicuculline; 12 with bicuculline). Raw electrophysiological data can be downloaded from the Collaborative Research in Computational Neuroscience (CRCNS) repository, available at http://dx.doi.org/10.6080/K00V89R6.

### Plasticity induction

Access resistance (R_a_) was monitored during the experiment; cells in which R_a_ changed by more than 20% were discarded from the analysis. As an additional stability requirement, the pre-pairing time series was fitted with a linear fit. Experiments with an unstable baseline in which significant trends were obtained with a linear fit (p<0.05) were discarded. After the pre-pairing epoch was complete (120 stimuli at 0.2 Hz), we used a pairing protocol for plasticity induction. The postsynaptic cell was (if necessary) switched from voltage to current clamp and a square current pulse was injected to produce a train of APs (typically 6–9 APs) 10 ms before the onset of the presynaptic stimulation (a 50 μs ECS pulse). This pairing was repeated 60 times at 0.1 Hz, after which the postsynaptic recording was switched back to voltage clamp (for V-clamp experiments), and testing of the connection resumed for at least an extra 10 minutes. The plasticity outcome of unitary connections after pairing was classified as LTP or LTD if we observed a statistically significant increase or decrease, respectively, in strength after pairing (t-test, p<0.05). Connections that did not reach statistical significance were classified as no change (NC); in keeping with prior results from our lab [16,17], those that did were classified as LTP or LTD if, in addition to reaching statistical significance, the magnitude of change exceeded an arbitrary threshold of 15% (+15% for LTP, -15% for LTD).

### Input mixing simulation

To compare the distribution of plasticity outcomes in SCS and ECS experiments, we used a Monte Carlo sampling procedure. A variable number of connections from our SCS database (previously reported in [17]; n = 42) were sequentially selected until their combined strengths were equal to or greater than 350pA (the typical size of ECS responses), at which point sampling was stopped. We called this the pre-pairing simulated compound response (SCR_pre_). A post-pairing simulated compound response (SCR_post_) was then calculated by adding the strengths of all selected pairs after they had been subject to a pairing protocol. The normalized difference between the SCR_pre_ and the SCR_post_, SCR Δ_N_ Strength, was then calculated. This procedure was repeated 26 times, to obtain a database of SCR Δ_N_ Strength similar in size to the observed differences in EPSC in the performed ECS experiments (ECS Δ_N_ Strength). To compare the variances of SCR Δ_N_ Strength and ECS Δ_N_ Strength we used an F-test. For illustration purposes, probability density functions of the outcomes were computed using density estimation with a standard Gaussian kernel. To simulate non-random mixing of unitary L4-L4 connections, a segregation parameter S with values [0,1] was introduced. When S = 0, input mixing is completely random, and the calculated probabilities of observing LTP/LTD/NC for each subsequently selected connection during sampling are the same as observed experimentally. When S = 1, inputs are completely segregated, so that the plasticity outcome of the first randomly selected connection (LTP/LTD/NC) determines whether the rest of the connections are randomly sampled from the LTP, LTD or NC subset of the data. Therefore, with S = 1 all selected connections for a given SCR show a qualitatively similar behavior in response to pairing (all potentiate, depress, or do not change). Between S = 0 and S = 1, the probability of obtaining a similarly behaving connection increase linearly. All the Δ_N_ Strength distributions are presented as probability density functions (PDFs) obtained by density estimation with a Gaussian kernel. All simulations were performed in R 2.71 (R Development Core Team; Foundation for Statistical Computing).

## Results

### Dataset

We analyzed the plasticity outcomes in response to a Hebbian pairing protocol of L4 excitatory cell afferents using ECS in the absence (n = 28; n = 14 in V-clamp, n = 14 in I-clamp) and presence (n = 26, I-clamp) of 2μM bicuculline in the bath.

### Extracellular stimulation

**Fig 1:**
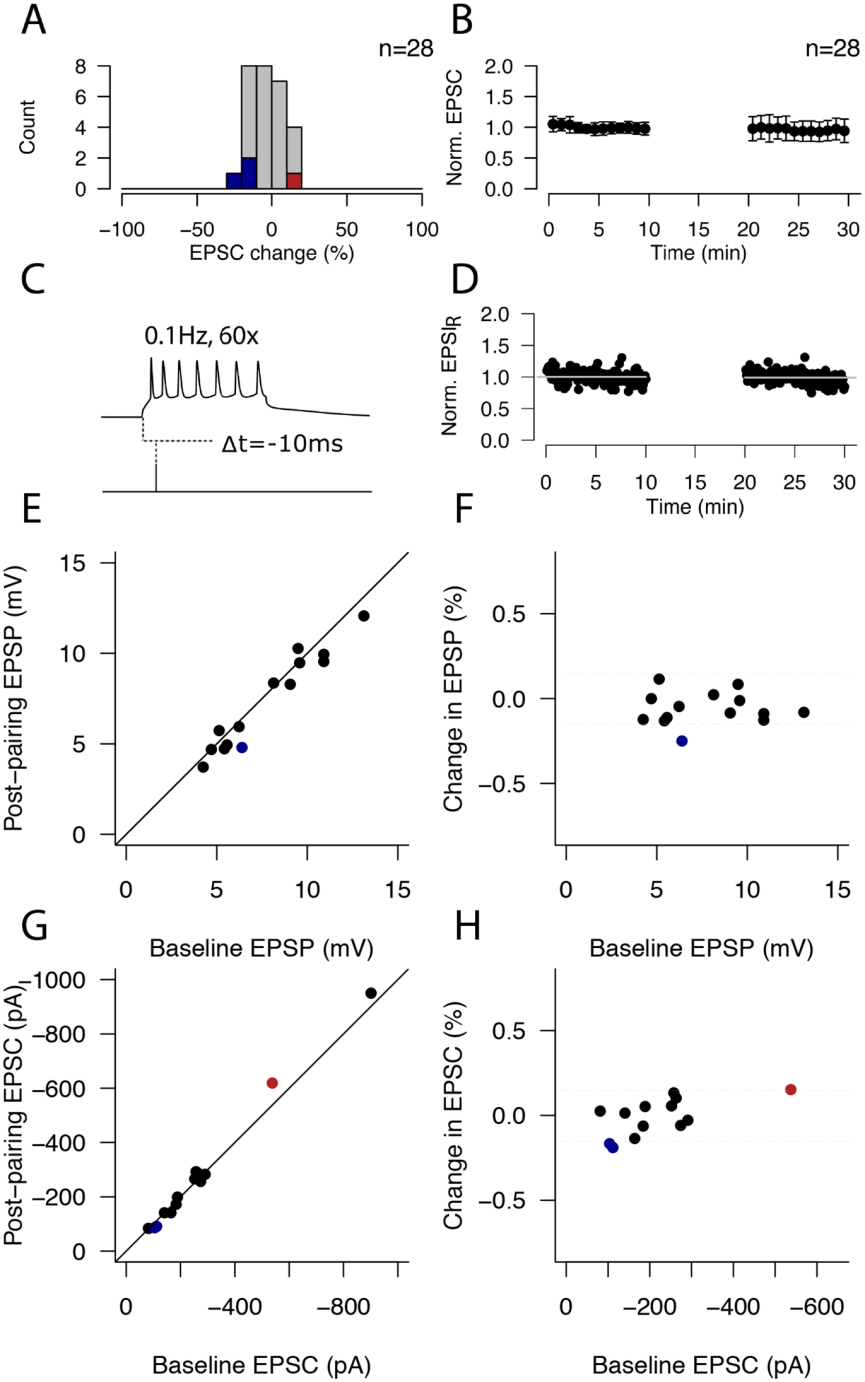
Extracellular stimulation of L4 afferents with intact inhibition results in limited or no plasticity. **(A)** ECS pairing protocol. A single 50μs ECS pulse was delivered 10ms after the onset of a postsynaptic burst of 6–9 action potentials triggered by intracellular current injection; the pairing was repeated 60 times at 0.1Hz. **(B)** Average normalized timeplot of all ECS experiments. The average change in peak response amplitude was -3.53±10.26%. Points correspond to minute averages; bars represent SEM. **(C)** Histogram showing the distribution of plasticity outcomes for 28 ECS pairing experiments. Plastic changes are defined as a >15%, significant (p<0.05) change in excitatory post-synaptic response (EPSR). LTD cells (n = 3/28) are shown as blue bars; a single LTP cell (n = 1/28) is shown as a red bar; grey bars indicate cells whose strength did not change after pairing (n = 24/28). **(D)** Example I-clamp ECS experiment that resulted in NC. Average pre- and post-pairing EPSP peak amplitudes are shown as grey lines. **(E)** Scatterplot showing the pre- and post-pairing amplitudes for all I-clamp experiments in the absence of bicuculline. **(F)** Scatterplot showing lack of correlation between the initial recorded synaptic strength under I-clamp (EPSP) and the plasticity outcome (p>0.9). **(G-H)** As (E-F), for V-clamp experiments (p>0.1). Grey dashed lines indicate plasticity threshold (±15% of initial synaptic strength).

We evaluated plasticity within L4 using conventional extracellular stimulation (ECS) of L4 afferents and applying a Hebbian pairing protocol that induces robust plasticity in visual cortical slices [16,17] and comparing the size of the evoked responses before and after pairing. For individual cells, plasticity (LTP and LTD) was defined as a change in the PSR that exceeded an arbitrary threshold of 15% and where the change was statistically significant (Student’s t-test, p<0.05). Under these criteria, few cells underwent LTP (n = 1/28, red in Fig 1A) or LTD (n = 3/28, blue in Fig 1A); overall the pairing protocol resulted in no net plasticity of EPSPs (recorded under current clamp) or EPSCs (under voltage clamp) for the population of cells studied (n = 28; p>0.8, Student’s t-test, Fig 1A). The net change in the compound postsynaptic response (PSR) was -5.67±9.47% in V-clamp and -1.41±10.92 in I-clamp. We did not observe a difference between V-clamp and I-clamp outcome distributions: both were Gaussian (p = 0.79 and p = 0.85 respectively, Shapiro-Wilks test) and their means and variances did not differ (p = 0.28, Student’s t-test; p = 0.61, Fisher’s F-test, respectively). Therefore, we combined both data sets for subsequent analysis (combined excitatory postsynaptic response, EPSR change = -3.8±34.3%). The normalized timeplot for the combined dataset is shown in Fig 1B (60 second bins; n = 28). Fig 1C shows an example pairing trial; the pairing protocol consisted of a 10-minute epoch 60 repetitions of this trial at 0.1Hz, and the relative timing of the pre- and postsynaptic activation was conserved across experiments (post leading pre by 10ms). The postsynaptic activation consisted of a square current injection that resulted in 6–9 action potentials. Fig 1D shows a representative example I-clamp experiment in which no plasticity was induced (EPSP change = -1±0.1%, p = 0.48, Student’s t-test). We did not observe a dependency of plasticity outcomes on initial synaptic response for either the IC (Fig 1E and 1F) or VC experiments (Fig 1G and 1H; both p>0.1). Thus, these results indicate that pairing of postsynaptic spiking with presynaptic activation in the presence of an intact inhibitory network mostly failed to induce plasticity in connections onto postsynaptic L4 neurons.

### Extracellular stimulation in the presence of 2μM bicuculline

**Fig 2:**
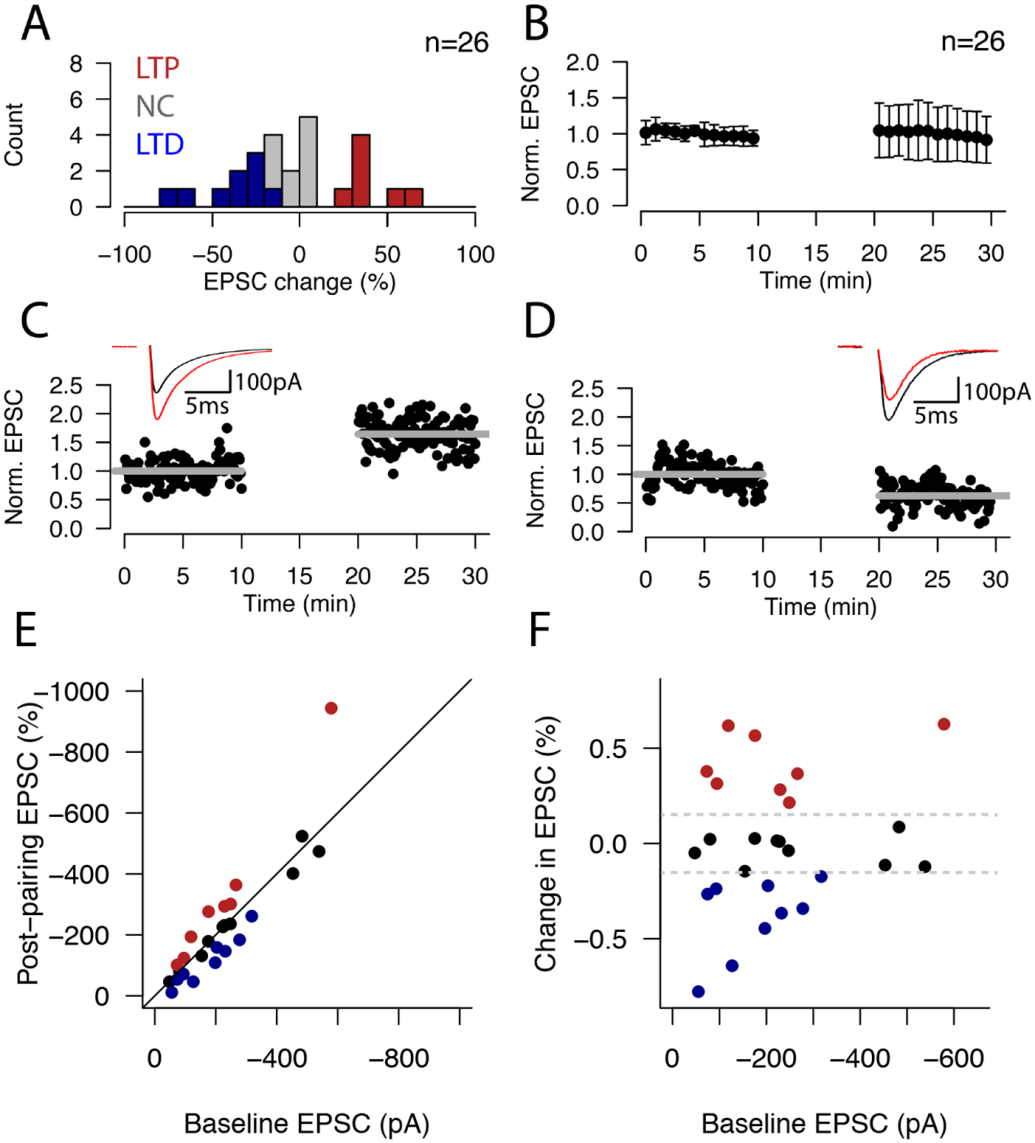
Extracellular stimulation of L4 afferents in the presence of 2μM bicuculline results in variable plasticity outcomes. **(A)** Histogram showing the distribution of plasticity outcomes for 26 ECS pairing experiments with bath application of 2μM bicuculline. LTD cells (n = 9/26) are shown as blue bars; LTP cells (n = 7/26) are shown as red bars; grey bars indicate cells whose strength did not change after pairing (n = 10/26). **(B)** Average normalized timeplot of all 26 ECS experiments performed with bath application of 2μM bicuculline, a GABA_A_R antagonist. The average change in peak response amplitude was -3.8±34.3%. Points correspond to minute averages; bars represent SEM. **(C)** Example ECS experiment that resulted in LTP in the presence of 2μM bicuculline. Average pre- and post-pairing EPSC peak amplitudes are shown as grey lines. Inset shows the average pre- (black trace) and post-pairing (red trace) responses. **(D)** Same as (C), for a representative experiment that resulted in LTD. **(E)** Scatterplot showing the pre- and post-pairing amplitudes for all bicuculline experiments (red = LTP, blue = LTD, black = NC). **(F)** Scatterplot showing lack of correlation between the initial recorded synaptic strength (EPSC) and the plasticity outcome (p>0.3). Grey dashed lines indicate plasticity threshold (±15% of initial synaptic strength).

To test the role of synaptic inhibition in controlling plasticity induction in L4-L4 synapses, we performed a second set of experiments in which we combined an identical plasticity induction paradigm with bath application of 2μM bicuculline to block GABA_A_R mediated inhibition (Fig 2, n = 26). Under these conditions, a greater proportion of cells underwent changes in synaptic response after pairing (16/26, or 63%). There was heterogeneity in the sign of the plasticity outcome, with 7/26 (27%) of cells undergoing LTP (average LTP EPSC change = +39.9±14.7%) and 9/26 (35%) of cells undergoing LTD (average LTD EPSC change = -38.5±20.4%). In a third subset of cells, no significant change in the evoked EPSC occurred (10/26 or 38.5%; average change = -3.0 ±7.6%) (Fig 2A). Interestingly, whereas the mean EPSC change of all experiments (n = 26) was not different in the presence (-3.8±34.3%; average time plot shown in Fig 1B) or absence (-3.5±10.3%; average time plot shown in Fig 2B) of bicuculline (p = 0.97, Student’s t-test), the variance of synaptic plasticity changes was significantly increased in bicuculline compared to control (p<0.01, Fisher’s test), reflecting the broadened distribution of plasticity outcomes (Fig 2A). Time plots from representative example cells that underwent LTP and LTD in the presence of bicuculline are shown in Fig 2C and 2D, respectively; insets in Fig 2C and 2D show the average EPSC trace in the pre-pairing (black) and post-pairing (red) epochs. For these connections, the change in strength was +59.4±26% for the LTP case (p<0.001, Student’s t-test; Fig 2C) and -37.6±19.9% for the LTD case (p<0.001, Student’s t-test; Fig 2D). We did not observe a dependency of plasticity outcomes on initial synaptic response (Fig 2E and 2F; p>0.5). These data show that, when multiple inputs onto a postsynaptic L4 cell are simultaneously activated in the absence of synaptic inhibition, they respond with differential polarity of responses (LTP, LTD or NC) to a pairing protocol, similar to what happens with single cell pairing in L4-L4 connections [17].

### Mixing of unitary L4-L4 inputs

**Fig 3:**
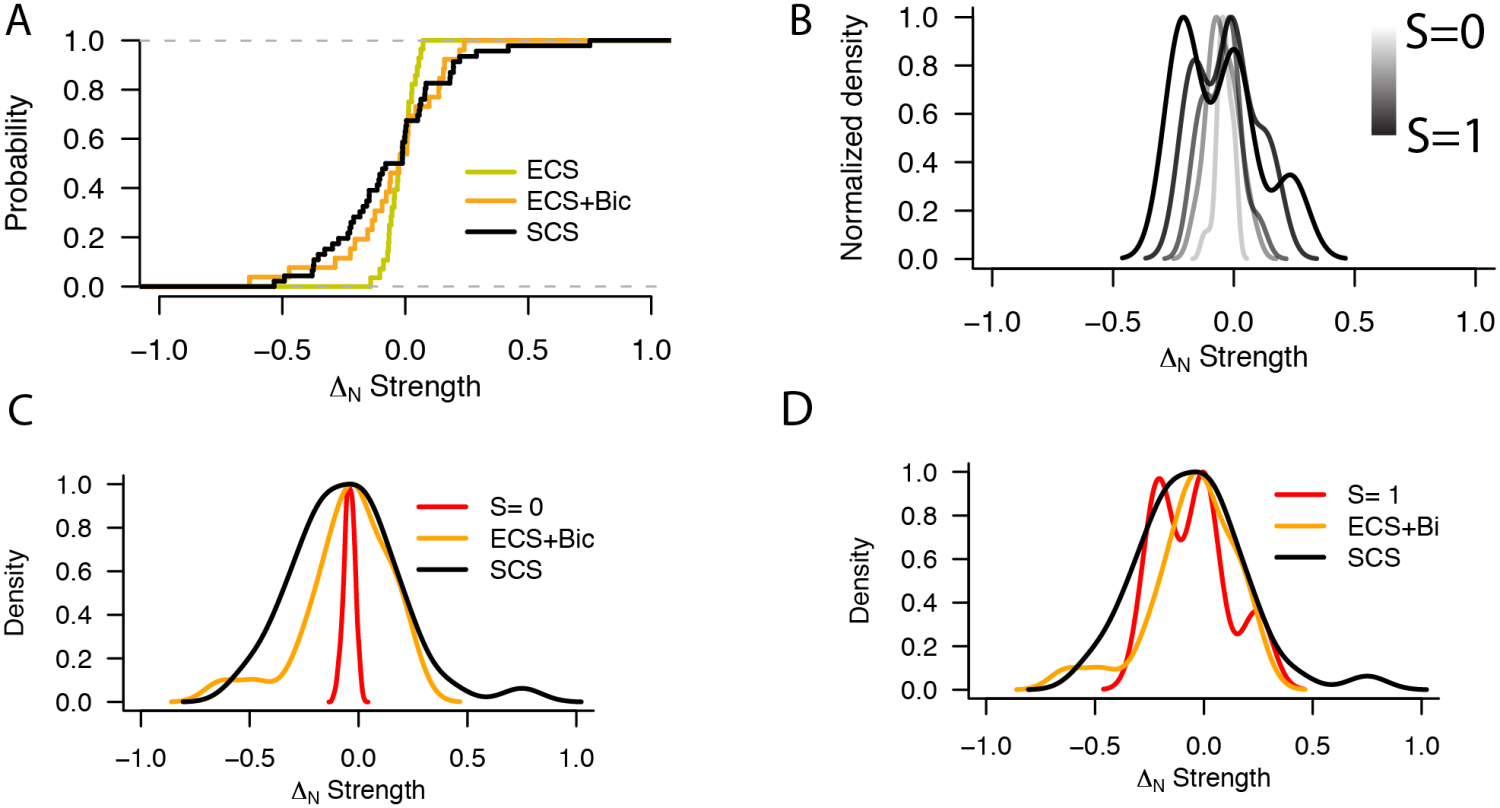
Comparison of ECS plasticity outcomes with mixing of SCS plasticity outcomes. **(A)** Cumulative distribution plots for normalized strength change in SCS (black), ECS (yellow) and ECS with 2μM bicuculline (green) experiments. **(B)** Normalized PDFs of SCR Δ_N_ Strengths (n = 26) for simulations with increasing segregation values, from random mixing (S = 0, lightest grey) to complete segregation by plasticity state (S = 1, black). **(C)** Normalized PDFs for SCS (black), ECS (yellow) and simulated random mixing SCRs (red, S = 0) Δ_N_ Strength distribution. PDFs have been normalized to the maximum (peak) value for clarity. **(D)** Normalized PDFs for SCS (black), ECS (yellow) and example simulated mixing SCRs (red) Δ_N_ Strength distribution with total segregation of inputs by plasticity state (S = 1).

Finally, we set out to compare the distribution of pairing-induced plasticity outcomes for the ECS stimulation paradigm with and without blockage of GABAergic inhibition (Figs 1 and 2) and our previously published single-cell stimulation (SCS) experiments [17]. These three datasets are summarized in the cumulative distribution plots in Fig 3A. The cumulative distributions are different when the plasticity outcomes from SCS experiments were compared to the ECS experiments performed in regular aCSF (green line; p<0.001, Kolmogorov-Smirnov test), but not when compared to the ECS experiments with bath application of 2μM bicuculline (yellow line; p = 0.16, Kolmogorov-Smirnov test). Since L4-L4 cell pair excitatory synaptic connections can potentiate or depress in response to a similar pairing protocol [17], we hypothesized that random mixing of synapses with different plasticity properties would result in little or no net plasticity in the postsynaptic cell, assuming no non-linearities. To test this idea, we constructed simulated compound responses (SCRs) by randomly selecting variable numbers of L4-L4 inputs (average number of inputs = 18.05±6.19) from our SCS database. This pre-pairing SCR (SCR_pre_) is therefore similar in concept to an ECS stimulation of L4 inputs only. A post-pairing SCR (SCR_post_) is then constructed in a similar fashion by summing the combined post-pairing strengths of all the previously selected SCS experiments (see Methods for details). By comparing this SCR_post_ with the combined strength of all selected connections in the pre-pairing condition, SCR_pre_, we obtained a SCR Δ_N_ Strength that expresses the resultant plasticity if the selected inputs had been simultaneously and independently activated. We repeated this procedure (n = 26, similar to the number of ECS experiments) and obtained a distribution of SCR Δ_N_ Strengths whose variance we compared to that of the ECS (n = 26) and SCS (n = 43) experiments (see Methods). We found that the distribution of SCR Δ_N_ Strengths was significantly narrower than that of either ECS (p<0.001, Fisher’s F-test) or SCS (p<0.001, Fisher’s F-test) experimental data (Fig 3C). Thus, random, independent mixing of unitary connections with different plasticity properties would not result in an overall plasticity change, contrary to what we observed in the ECS+bicuculline experiments (Fig 2A). We therefore tested the hypothesis that a plasticity outcome distribution similar to ECS+bicuculline would be obtained if all inputs onto a single postsynaptic state were in a similar synaptic state, and thus respond similarly to the induction protocol. To do this we obtained a new distribution of SCR Δ_N_Strengths with the addition of a segregation parameter S, whose value ranges from 0 (random mixing; cartoon in Fig 3B, left) to 1 (complete segregation of inputs by plasticity state; Fig 3B, right—see Methods) in 0.1 increments. When S = 0, mixing of synaptic inputs with different plasticity properties is completely random, no biases are imposed and the probability of sampling a connection with a given plasticity outcome corresponds to that observed experimentally (i.e. 7/43 or 16.27% for LTP and 18/43 or 41.86% for both NC and LTD; data from [17]). For S = 1, segregation is complete, and all selected inputs for a given SCR will be drawn only from the SCS LTP, NC or LTD data subsets. For 0<S<1, there is a progressive linear biasing of the LTP/NC/LTD sampling probabilities, such that subsequent samples are preferentially selected to have a similar outcome as the first (randomly drawn) sample. With increasing values of S, the distribution of observed outcomes progressively broadened (Fig 3B). The superimposed PDFs for the distribution of Δ_N_ Strength for SCS, ECS and SCR with S = 0 are shown in Fig 3C; note the narrower PDF corresponding to the SCR compared to that for S = 1 in Fig 3D. We then compared the variances of the SCRs for every S value with that of the ECS experiments in the presence of bicuculline. We found that for most S values, the variance of the SCRs is lower (p≤0.05, Fisher’s F-test); only for values of S≥0.9 was there no significant difference in variance between the SCR and ECS Δ_N_ Strength distributions (p>0.05, Fisher’s F-test). Thus, if segregation of inputs by plasticity state were the explanation for the observed ECS+bicuculline plasticity distribution this segregation would need to be almost complete.

## Discussion

### Summary

We studied the plasticity of L4 inputs onto L4 excitatory neurons in response to pairing of pre- and postsynaptic activity in the absence (n = 28, Fig 1A) and presence (n = 26, Fig 2A) of a GABA_A_R antagonist drug (2μM bicuculline). In both cases, the average change across all experiments was negligible (<4% in both cases), but a case-by-case examination of the plasticity outcomes revealed profound differences. With intact inhibition, a very small fraction of cells underwent a plastic change by our criteria (n = 4/28; LTP in 1/28 and LTD in 3/28 cells). This proportion was considerably increased when inhibition was abolished (n = 16/26; LTP in 7/26 and LTD in 9/26 cells), demonstrating that inhibitory activity limits induction of synaptic plasticity in connections onto L4 cells in V1. These outcomes of plasticity could be partially, but not fully, explained by segregation of inputs in different plasticity states onto postsynaptic L4 neurons.

### GABA_A_-mediated inhibitory activity limits plasticity expression within L4

ECS within L4 is likely to activate a diverse population of excitatory and inhibitory inputs to neighboring L4 cells [3,19]. Activation of synaptic inhibition within L4 by ECS of deeper cortical layers or cortical white matter restricts plasticity at L4 to L2/3 synapses through an inhibitory gating mechanism [12,20]. Similar to what happens in the case of projections onto supragranular layers [15,18], GABA_A_-mediated inhibitory activity within L4 limits the induction of synaptic plasticity in intralaminar excitatory connections (Figs 1A and 2A). Activation of shunting inhibition in the postsynaptic membrane could reduce the amplitude or spread of synaptic depolarization or impede the backpropagation of action potentials from the soma to sites of synaptic input in the dendritic tree [21,22]. Both mechanisms would result in a reduction in the amount of Ca^2+^ entry through NMDARs and VGCCs to the postsynaptic cell. Given the necessary role of Ca^2+^ for induction of synaptic plasticity [23,24], this mechanism may reduce the expression of LTP and LTD in the presence of intact inhibition. In addition, GABAergic inhibitory cells within L4 are electrically coupled via gap junctions [25] and therefore widespread activation of the L4 inhibitory network may act as a circuit-wide impediment to the modification of synaptic weights, both of intralaminar connections within L4 and in connections originating in L4 and targeting supragranular (L2/3) cortical areas [12,15].

### Plasticity of different signs is elicited when blocking GABA_A_ receptors

When GABA_A_-mediated inhibition is removed, plasticity in response to a pairing protocol can be of different signs; the pairing protocol can result in LTP, LTD or no change outcomes (7/26, 9/26 and 10/26, or approximately 27%, 34.5% and 38/5% of connections in our dataset; Fig 2). Furthermore, we show that the plasticity outcome is not related to the baseline strength of the connection (Fig 2E and 2F). We have previously reported similar variability in plasticity responses to a single pairing protocol in V1 using ECS in L4-L2/3 connections and using SCS in L4-L4 connections [16,17]. Spike-timing dependent plasticity (STDP), in which simultaneous activation of pre- and postsynaptic activity results in modification of synaptic weights, is a possible mechanism whereby plasticity changes arise in our preparation. STDP with a single postsynaptic spike occurs in a variety of brain regions including hippocampus [26], optic tectum [27], and neocortex [28–30]. Here, the timing of pre- and postsynaptic activation is consistent across experiments, with the postsynaptic activation leading the presynaptic stimulation pulse by 10ms (see Fig 1A and methods). Thus, the relative timing of presynaptic and postsynaptic activity cannot be responsible for the induction of plasticity of different signs via STDP mechanisms, in which differences in timing between the pre- and postsynaptic activation give rise to different plasticity outcomes [27]. Other explanations for the variable outcomes of plasticity include differences in postsynaptic calcium handling mechanisms [16], putative different subclasses of excitatory neurons [31,32], different developmental stages of L4 neurons [33,34], ECS-mediated differential excitation of neuromodulatory fibers which are known to modulate plasticity induction [20], ECS-mediated stimulation of different kinds of afferents which may have different synaptic transmission and plasticity properties (e.g. from thalamocortical fibers or infragranular layers [35,36]), differential involvement of other components of the inhibitory network (see below) or sensitivity to the initial state of the connection, such that its initial properties determine or bias the outcome to the pairing protocol [17,37,38]. This last mechanism may play a homeostatic role in V1 by preventing saturation of synaptic weights and keeping V1 synapses within a functional boundary of synaptic weights that does not compromise its role in processing early visual information. Similarly, the developmental time course of GABAergic networks, which are excitatory early in development and later become inhibitory [39] would permit synaptic modification during early postnatal development and limit further plasticity after the developmental switch to inhibition.

### Regulation of synaptic plasticity by inhibitory networks

The data here presented demonstrate the role of ionotropic (GABA_A_) receptors in limiting synaptic plasticity in L4 neurons. GABA_B_-mediated synaptic inhibition, however, was intact and may have contributed to the expression of synaptic plasticity in our system. Recent reports have shown that potentiation of inhibitory connections (LTPi) through a GABAB–Gi/o-dependent potentiation of GABAA-mediated IPSPs is a powerful modulator of synaptic plasticity expression in visual cortex [40]. Since GABA_B_-mediated inhibition is intact, our experiments cannot rule out LTPi induction through activation of a GABA_B_–Gi/o cascade; however, given our pharmacological block of GABA_A_ receptors, it is unlikely that these changes would be manifested through a potentiation of GABA_A_ IPSPs, but other signaling pathways associated with GABA_B_-Gi/o, such as modulation of voltage gated calcium channels or potassium conductances may play an important role in shaping plasticity [40].

### The role of disinhibition in ocular dominance plasticity

Down-regulation of GABAergic inhibitory networks plays an important role in ocular dominance plasticity (ODP), an important model of postnatal synaptic plasticity [2,8,41] in which removal of visual input causes robust long-term changes in cortical circuitry, including layer 4 [34,42]. Ocular deprivation causes an increase in excitation accompanied by a decrease in inhibition in cortical circuits [34]. Recent results have shown that a critical step in the progression of ODP is an initial reduction of excitatory drive onto fast-spiking interneurons, which subsequently reduces feedback inhibition onto excitatory neurons and allows for plasticity induction and a restoration of evoked firing rates [43,44]. Our results are entirely consistent with such a mechanism; indeed, our pharmacological block of GABAA receptors may mirror the *in vivo* process through a similar reduction in inhibition, which is then followed by synaptic plasticity induction triggered by paired pre- and postsynaptic activity. The current results provide a closer look at the expression mechanism of these plastic changes at a synaptic level (LTP/LTD).

In summary, both inhibitory limitation of GABA_A_-mediated synaptic plasticity and homeostatic dependence of plasticity outcome on initial synaptic characteristics would limit the amount of synaptic plasticity in connections arising from L4 in the adult V1, perhaps as a means of maintaining the functional characteristics of these connections in the mature brain.

### Mixing of synaptic inputs in different states

Interestingly, the variable outcome in synaptic plasticity with a single induction protocol we observe here was similar to our previous reports using single L4 cell stimulation [17]. However, our simulations indicate that the observed plasticity outcomes under ECS are unlikely to result from the accumulation of independent changes of combinations of unitary connections (Fig 3). A possible explanation for this discrepancy is that the common history of activation of the postsynaptic neuron might confer some similarities in synaptic state to incoming connections stimulated with ECS, and that these similarities may result in a partial coordination of plasticity outcomes across all synapses. Other factors could result in a common postsynaptic plasticity state, such as a common gene expression profile or similar phosphorylation patterns of proteins involved in plasticity signaling cascades [31,32]. Our simulations suggest that the variability of plasticity outcomes with ECS could potentially be explained by almost complete segregation of L4-L4 connections in different plasticity states (i.e. a predominance of inputs that undergo either LTP, LTD or NC in response to pairing). However, previous results show that multiple unitary inputs onto a single postsynaptic cell can indeed undergo plasticity of different sign [17], and thus this is unlikely to be the only factor at play. A simple alternative explanation would be that plasticity induction resulting from ECS does not result from a linear combination of multiple unitary synaptic plasticity events; indeed, multiple non-linearities in synaptic transmission exist such as the generation of dendritic calcium spikes, voltage filtering and the location of synapses along the dendritic arbor of the postsynaptic neuron, which will cause slight differences in timing of the back-propagated AP (although some of these are at play in unitary connections, for example, unique synaptic contacts between pairs of neurons can be located in very different locations in the postsynaptic dendritic arbor [35,45]). An additional factor at play is the modulation of synaptic plasticity expression by GABA_B_-mediated inhibition. Previous reports have shown that long-term modulation of inhibitory synaptic transmission is an important modulator of visual cortical synapses, and GABA_B_-mediated modulation of inhibition triggers plasticity of inhibitory synapses and directly regulates the sign of plasticity at excitatory synapses [40]. A similar mechanism may be at play here, and contribute to the heterogeneity in synaptic plasticity outcomes observed in extracellular stimulation experiments. Finally, these results do not take into account possible modification of inhibitory connections, which are known to undergo plasticity of different sign themselves and modulate plasticity of excitatory connections [34,40,46]. An attempt to provide a complete characterization of the plasticity properties of the cortical network in V1 will have to include these additional processes which where not included in our simulations. Further experiments, for example examining the plasticity outcome after stimulating multiple presynaptic neurons onto a single postsynaptic targets [35], will be necessary to address the relative contribution of the postsynaptic target identity versus other factors influencing plasticity outcomes.

## Conclusion

Here we demonstrate that GABA_A_-mediated inhibition plays an important role in limiting synaptic plasticity induction within L4 of V1, similarly to what happens in L4 outputs to supragranular layers, in a process that may be related to synaptic plasticity facilitated by disinhibition in *in vivo* plasticity. Furthermore, L4-L4 connections can respond differentially (LTP/no change/LTD) to a common plasticity induction protocol, in a mechanism that may be influenced by the state or common history of the postsynaptic cell. We hypothesize that segregation of connections with similar plasticity profiles may play a role in determining this plasticity profile, and that the combination of these mechanisms serves to limit the modification of synapses within L4 to maintain an efficient network for early processing of visual information in post-critical period networks.
